# Soil ingestion among young children in rural Bangladesh

**DOI:** 10.1038/s41370-019-0177-7

**Published:** 2019-10-31

**Authors:** Laura H. Kwong, Ayse Ercumen, Amy J. Pickering, Leanne Unicomb, Jennifer Davis, James O. Leckie, Stephen P. Luby

**Affiliations:** 1 Woods Institute for the Environment, Stanford University, Stanford, CA, USA; 2 Department of Forestry and Environmental Resources, North Carolina State University, Raleigh, NC, USA; 3 Department of Civil and Environmental Engineering, Tufts University, Medford, MA, USA; 4 International Centre for Diarrhoeal Disease Research, Bangladesh, Dhaka, Bangladesh; 5 Department of Civil and Environmental Engineering, Stanford University, Stanford, CA, USA

**Keywords:** Soil, Dust, Geophagia, Bangladesh, Extraction efficiency

## Abstract

Ingestion of soil and dust is a pathway of children’s exposure to several environmental contaminants, including lead, pesticides, and fecal contamination. Empirically based estimates of central tendency for soil consumption by children in high-income countries range from 9 to 135 dry mg/day. Using a Monte Carlo simulation, we modeled the mass of soil directly and indirectly ingested per day by rural Bangladeshi children and identified the parameters that influence the mass ingested. We combined data from observations of direct and indirect ingestion among children with measurements of soil mass on the children’s hands, mother’s hands, and objects to quantify soil ingestion/day. Estimated geometric mean soil ingestion was 162 dry mg/day for children 3–5 months, 224 dry mg/day for children 6–11 months, 234 dry mg/day for children 12–23 months, 168 dry mg/day for children 24–35 months, and 178 dry mg/day for children 36–47 months old. Across all age groups, children placing their hands in their mouths accounted for 46–78% of total ingestion and mouthing objects contributed 8–12%. Direct ingestion of soil accounted for nearly 40% of soil ingested among children 6–23 months old. Sensitivity analyses identified that the parameters most affecting the estimates were the load of soil on the child’s hand, the frequency of hand-to-mouth contacts while not eating, and, for children 6–23 months old, the frequency of direct soil ingestion. In a rural, low-income setting, children’s soil consumption was substantially more than the estimates for children in high-income countries. Further characterizing soil ingestion of children in low-income contexts would improve assessments of the risks they face from soil-associated contaminants.

## Introduction

Ingesting soil and dust are primary pathways of children’s exposure to several environmental contaminants. Quantifying the amount of soil and dust children ingest has been used to assess their exposure to heavy metals such as lead [1], high doses of which may result in anemia, neurobehavioral toxicity, and death [2]. Soil and dust ingestion is also an important pathway for intake of pesticides [3], which can be carcinogenic and neurotoxic [4]. Soil ingestion by young children has also been associated with markers of environmental enteric dysfunction and growth faltering [5]. Quantifying children’s exposure to such environmental contaminants through accurate estimation of their soil and dust ingestion supports regulation of environmental contaminants to limit the risk of adverse effects [6].

Methodologies commonly used to estimate soil and dust ingestion include tracers, biokinetic model comparison, and activity pattern methods. The tracer method uses a mass balance approach and has several limitations, as tracers may be ingested from unmeasured sources or excreted in unmeasured media [7]. The biokinetic model comparison method compares direct biomarker measurements with modeled biomarker levels due to ingestion, inhalation, and dermal exposure. Individual model parameters are difficult to validate because there are multiple model specifications and combinations of parameter values that could result in agreement between model estimates and measured biomarker levels [8].

The activity pattern methodology considers the frequency of children’s activities associated with ingestion of and contact with soil and dust and the amount of soil and dust transferred with each of these contacts. Early activity pattern studies modeled only indirect ingestion and used nonempirical estimates of the frequency of contacts with hands and objects [9–12]. More comprehensive models incorporated empirically observed hand-to-mouth and object-to-mouth contact frequencies and account for incomplete transfer of soil residues from surfaces into the mouth, with resulting arithmetic mean estimates for soil and dust ingestion of 68 dry mg/day among U.S. children [7] and 61 dry mg/day among Canadian children [13]. One difficulty with the activity pattern method is the limited empirical data on both the frequency of children’s contacts and the soil transfer efficiency rates, especially in low-income countries [14, 15]. Additionally, the lack of quantitative studies on the prevalence and frequency of direct soil consumption by children has led to direct ingestion previously excluded as a pathway of soil intake.

Estimates of soil and dust ingestion by children in high-income countries vary by orders of magnitude, depending, in part, on the methodology used to determine the estimates [16]. These estimates may also differ due to contextual, seasonal, and cultural factors associated with the study population. For example, young children in the Netherlands who were camping had higher estimated ingestion rates than children who were not camping, and kindergarteners had higher rates during the summertime than during the school year [17].

There is reason to suspect that children in low-income countries may have substantially higher rates of soil and dust ingestion than children in high-income countries. In low-income countries, homes more often have floors made of earth, rather than materials such as tile or hardwood that can be more thoroughly cleaned to reduce dust exposure. Children may play outdoors on bare soil rather than on pavement or grass. Children from low-income countries may also have different rates of placing soil-laden hands or objects in their mouths and directly consuming soil. We have previously found that rural Bangladeshi children have markedly higher rates of placing hands and objects in their mouths compared U.S. children [18, 19]. Two studies have estimated the amount of soil and dust ingested by children in low-income countries using limited empirical data: one study reported 8–108 g soil ingested/day based on self-reports by children 5–18 years old [20], while the other estimated a one-year-old ingests 21 wet g/day based on the assumption that when children put soil in their mouths, their hands were as contaminated as if soil was poured onto the outstretched hand from above [21]. To more accurately estimate soil and dust ingestion among children in low-income countries, we modeled the mass of soil and dust ingested by rural Bangladeshi children 3–47 months old using the activity pattern method, combining observations of direct and indirect ingestion with empirical estimates of soil mass on hands and objects.

## Methods

### Empirical measurements of model input parameters

We used structured and video observations, anthropometric data, and measurements of soil mass and fecal contamination on hands and objects collected from Bangladeshi participants enrolled in WASH Benefits, a large, cluster-randomized trial of water, sanitation, and hygiene interventions [22]. These data are described in the SI.

### Model overview

We used a probabilistic Monte Carlo simulation to model the amount of soil ingested by a rural Bangladeshi child every waking hour of a single day (Eq. 1). In this manuscript, we refer to both soil and dust (soil that is indoors [7]) as “soil”. We did not distinguish between soil and dust because the frequency of hand-to-mouth contact, object-to-mouth contacts, and direct soil ingestion by children in this study was not significantly different between indoor and outdoor locations [19]. Hence, the ratio of soil to dust ingestion is approximated by the ratio of time children spend outdoors, which ranged from 48 to 63% for children in different age groups [19].

Using age-specific distributions for relevant parameters, our model estimates soil ingestion due to placing soiled hands (Eqs. 2–4) and objects (Eq. 5) into the mouth for each hour a child is awake. We used our empirical measurements to estimate the load of soil on hands and objects and multiplied by the hand- or object-to-mouth transfer efficiency related to the type of mouthing: contacts involving the hand entering the mouth (an oral contact) or the hand touching only the lips or outside of the mouth (a peri-oral contact) [23]. We also considered the frequency of oral and peri-oral hand- and object-mouthing. We simulated variations in a child’s day by summing as many selected hourly sub-totals as the child is awake (awake_c,*i*_), plus one more hourly value multiplied by the fractional hour the child was awake, rounded to the nearest tenth. For example, if a child was awake for 11.4 h, we add 11 of the hourly results, plus 0.4 times the next hourly results. We then added the amount of soil ingested from direct soil consumption, calculated as mass per day (Eq. 6). Mother and child hand surface area were held constant for each one-day simulation. Child-specific parameters are denoted with the subscript “c”, followed by a number that represents the child’s age group (*i*), corresponding to age windows based on U.S. EPA exposure assessment guidelines [24]. Caregiver-specific parameters are denoted with the subscript “m”. Parameter abbreviations, descriptions, and values are given in Table 1.

**Table 1:** Parameters and associated distributions for modeling soil ingestion rates among young children in rural Bangladesh

Parameter symbol	Parameter	Units	Age group	Form	v1	v2	Min	Mean	Median	Max	Reference
HMTE	Hand-to-mouth transfer efficiency	—	—	beta	5.20	2.6	0.11	0.67	0.69	0.99	This study
PTE	Peri-oral transfer efficiency from the hand to the lips to the mouth	—	—	beta	5.20	2.6	0.00	0.30	0.28	0.93	IOM [26]
SOIL_c_	Load of on child’s one hand	dry mg/cm^2^	—	empirical			0.00	0.10	0.07	0.95	This study
SA_hand,c,4_	Surface area of child’s one hand	MPN/hand	3–5 months	empirical			74.59	98.31	98.52	119.18	This study
SA_hand,c,9_	Surface area of child’s one hand	MPN/hand	6–11 months	empirical			75.52	106.17	105.85	140.20	This study
SA_hand,c,18_	Surface area of child’s one hand	MPN/hand	12–23 months	empirical			80.01	121.18	121.07	177.38	This study
SA_hand,c,30_	Surface area of child’s one hand	MPN/hand	24–35 months	empirical			96.82	127.53	126.64	166.87	This study
SA_hand,c,36_	Surface area of child’s one hand	MPN/hand	36–47 months	empirical			139.63	146.47	145.84	153.28	This study
HM.nd_c,4_	Frequency of child’s hand-to-mouth contact while not eating	times/h	3–5 months	empirical			2.26	17.60	11.70	59.13	This study
HM.nd_c,9_	Frequency of child’s hand-to-mouth contact while not eating	times/h	6–11 months	empirical			0.85	13.04	10.23	45.57	Kwong et al. [18, 19]
HM.nd_c,18_	Frequency of child’s hand-to-mouth contact while not eating	times/h	12–23 months	empirical			0.00	10.58	10.15	31.36	Kwong et al. [18, 19]
HM.nd_c,30_	Frequency of child’s hand-to-mouth contact while not eating	times/h	24–35 months	empirical			0.49	11.70	8.75	36.29	Kwong et al. [18, 19]
HM.nd_c,36_	Frequency of child’s hand-to-mouth contact while not eating	times/h	36–47 months	empirical			2.25	8.36	6.59	16.46	Kwong et al. [18, 19]
HM.d.episode_c,4_	Frequency of episodes when child feeds self by hand	episodes/h	3–5 months	empirical			0.00	0.01	0.00	0.25	Kwong et al. [18, 19]
HM.d.episode_c,9_	Frequency of episodes when child feeds self by hand	episodes/h	6–11 months	empirical			0.00	0.17	0.00	5.23	Kwong et al. [18, 19]
HM.d.episode_c,18_	Frequency of episodes when child feeds self by hand	episodes/h	12–23 months	empirical			0.00	1.58	0.48	6.36	Kwong et al. [18, 19]
HM.d.episode_c,30_	Frequency of episodes when child feeds self by hand	episodes/h	24–35 months	empirical			0.66	2.72	2.57	5.15	Kwong et al. [18, 19]
HM.d.episode_c,36_	Frequency of episodes when child feeds self by hand	episodes/h	36–47 months	empirical			1.55	3.99	2.90	8.28	Kwong et al. [18, 19]
HM.o_c_	Fraction of child’s hand-to-mouth contacts that are oral	—	—	empirical			0.00	0.50	0.58	0.89	This study
HF.o_c_	Fraction of child’s hand that contacts the mouth in an oral hand-to-mouth contact	—	—	empirical			0.06	0.19	0.19	0.63	This study
HF.po_c_	Fraction of child’s hand that contacts the mouth in a peri-oral contact	—	—	empirical			0.01	0.02	0.01	0.05	This study
SOIL_hand,m_	Load of soil on caregiver’s one hand	MPN/hand	—	empirical			0.00	0.03	0.02	0.11	This study
SA_hand,m_	Surface area of caregiver’s one hand	MPN/hand	—	empirical			252.44	321.46	318.58	452.09	This study
HM.nd_m,4_	Frequency of contacts between caregiver’s hand and child’s mouth	times/h	3–5 months	empirical			0.19	2.61	2.01	9.42	Kwong et al. [18, 19]
HM.nd_m,9_	Frequency of contacts between caregiver’s hand and child’s mouth	times/h	6–11 months	empirical			0.13	1.88	1.08	10.36	Kwong et al. [18, 19]
HM.nd_m,18_	Frequency of contacts between caregiver’s hand and child’s mouth	times/h	12–23 months	empirical			0.00	0.95	0.39	7.15	Kwong et al. [18, 19]
HM.nd_m,30_	Frequency of contacts between caregiver’s hand and child’s mouth	times/h	24–35 months	empirical			0.00	0.48	0.00	2.17	Kwong et al. [18, 19]
HM.nd_m,36_	Frequency of contacts between caregiver’s hand and child’s mouth	times/h	36–47 months	empirical			0.00	2.49	0.33	14.06	Kwong et al. [18, 19]
HM.d.episode_m,4_	Frequency of episodes when caregiver feeds child by hand	episodes/h	3–5 months	empirical			0.00	0.07	0.00	1.50	Kwong et al. [18, 19]
HM.d.episode_m,9_	Frequency of episodes when caregiver feeds child by hand	episodes/h	6–11 months	empirical			0.00	0.23	0.00	3.85	Kwong et al. [18, 19]
HM.d.episode_m,18_	Frequency of episodes when caregiver feeds child by hand	episodes/h	12–23 months	empirical			0.00	0.72	0.21	4.97	Kwong et al. [18, 19]
HM.d.episode_m,30_	Frequency of episodes when caregiver feeds child by hand	episodes/h	24–35 months	empirical			0.00	0.72	0.46	3.76	Kwong et al. [18, 19]
HM.d.episode_m,36_	Frequency of episodes when caregiver feeds child by hand	episodes/h	36–47 months	empirical			0.00	0.38	0.29	0.91	Kwong et al. [18, 19]
HF.o_m,4_	Fraction of caregiver’s hand mouthed in an oral contact	—	3–5 months	empirical			0.01	0.06	0.06	0.25	This study
HF.o_m,9_	Fraction of caregiver’s hand mouthed in an oral contact	—	6–11 months	empirical			0.01	0.06	0.06	0.28	This study
HF.o_m,18_	Fraction of caregiver’s hand mouthed in an oral contact		12–23 months	empirical			0.02	0.07	0.07	0.34	This study
HF.o_m,30_	Fraction of caregiver’s hand mouthed in an oral contact	—	24–35 months	empirical			0.02	0.08	0.07	0.36	This study
HF.o_m,36_	Fraction of caregiver’s hand mouthed in an oral contact	—	36–47 months	empirical			0.02	0.09	0.09	0.37	This study
HF.po_m,4_	Fraction of caregiver’s hand mouthed in a peri-oral contact	—	3–5 months	empirical			0.00	0.01	0.00	0.02	This study
HF.po_m,9_	Fraction of caregiver’s hand mouthed in a peri-oral contact	—	6–11 months	empirical			0.00	0.01	0.00	0.02	This study
HF.po_m,18_	Fraction of caregiver’s hand mouthed in a peri-oral contact	—	12–23 months	empirical			0.00	0.01	0.01	0.02	This study
HF.po_m,30_	Fraction of caregiver’s hand mouthed in a peri-oral contact	—	24–35 months	empirical			0.00	0.01	0.01	0.03	This study
HF.po_m,36_	Fraction of caregiver’s hand mouthed in a peri-oral contact	—	36–47 months	empirical			0.00	0.01	0.01	0.03	This study
SOIL_obj_	Load of soil on object	MPN/cm^2^	—	empirical			0.00	0.01	0.00	0.19	This study
OM_c,4_	Frequency of child’s object-to-mouth contacts	times/h	3–5 months	empirical			0.25	17.75	8.17	77.41	Kwong et al. [18, 19]
OM_c,9_	Frequency of child’s object-to-mouth contacts	times/h	6–11 months	empirical			0.33	19.01	12.67	115.87	Kwong et al. [18, 19]
OM_c,18_	Frequency of child’s object-to-mouth contacts	times/h	12–23 months	empirical			0.34	17.40	11.16	74.10	Kwong et al. [18, 19]
OM_c,30_	Frequency of child’s object-to-mouth contacts	times/h	24–35 months	empirical			1.46	24.07	18.83	58.70	Kwong et al. [18, 19]
OM_c,36_	Frequency of child’s object-to-mouth contacts	times/h	36–47 months	empirical			7.03	18.95	14.01	41.57	Kwong et al. [18, 19]
SAM_obj_	Mouthed surface area of object	cm^2^	—	exponential	0.11		1.00	9.98	7.38	49.83	Leckie et al. [23]
DIS_c,4_	Fraction of children directly ingesting soil	times/day	3–5 months	empirical			0.22	0.22	0.22	0.22	Kwong et al. [18, 19]
DIS_c,9_	Fraction of children directly ingesting soil	times/day	6–11 months	empirical			0.56	0.56	0.56	0.56	Kwong et al. [18, 19]
DIS_c,18_	Fraction of children directly ingesting soil	times/day	12–23 months	empirical			0.56	0.56	0.56	0.56	Kwong et al. [18, 19]
DIS_c,30_	Fraction of children directly ingesting soil	times/day	24–35 months	empirical			0.08	0.08	0.08	0.08	Kwong et al. [18, 19]
DIS_c,36_	Fraction of children directly ingesting soil	times/day	36–47 months	empirical			0.20	0.20	0.20	0.20	Kwong et al. [18, 19]
SM_c,4_	Frequency of direct soil ingestion among mouthers only	times/day	3–5 months	empirical			2.20	4.80	5.95	6.24	Kwong et al. [18, 19]
SM_c,9_	Frequency of direct soil ingestion among mouthers only	times/day	6–11 months	empirical			2.07	12.44	6.73	64.62	Kwong et al. [18, 19]
SM_c,18_	Frequency of direct soil ingestion among mouthers only	times/day	12–23 months	empirical			2.79	9.02	7.16	26.01	Kwong et al. [18, 19]
SM_c,30_	Frequency of direct soil ingestion among mouthers only	times/day	24–35 months	empirical			1.00	3.98	4.00	7.00	Kwong et al. [18, 19]
SM_c,36_	Frequency of direct soil ingestion among mouthers only	times/day	36–47 months	empirical			1.00	3.99	4.00	7.00	Kwong et al. [18, 19]
SW_DI_	Mass of soil ingested per direct ingestion event	dry mg	—	beta	5.30	158.6	0.01	0.03	0.03	0.07	This study
awake_c,4_	Duration awake	h/day	3–5 months	normal	13.60	2.1	6.20	10.40	10.39	14.60	Galland et al. SI ref. [28]
awake_c,9_	Duration awake	h/day	6–11 months	normal	12.90	1.3	8.20	11.15	11.15	14.60	Galland et al. SI ref. [28]
awake_c,18_	Duration awake	h/day	12–23 months	normal	12.60	1.3	8.80	11.42	11.43	14.00	Galland et al. SI ref. [28]
awake_c,30_	Duration awake	h/day	24–35 months	normal	12.00	1.2	9.80	12.01	12.01	14.30	Galland et al. SI ref. [28]
awake_c,36_	Duration awake	h/day	36–47 months	normal	12.00	1.2	9.80	12.02	12.01	14.30	Galland et al. SI ref. [28]

The subscript ‘c’ refers to children, while the subscript ‘m’ refers to caregivers. The number after the subscript refers to the child age group: 3–5 months (c = 4), 6–11 months (c = 9), 12–23 months (c = 18), 24–35 months (c = 30), and 36–47 months (c = 42)

(1) $${\text{SOIL}}_{\text{day}}=\overset{{\text{awake}}_{c,i}}{\underset{0}{\Sigma }}\left({\text{SA}}_{\text{hand},c,i}*({\text{SOIL}}_{\text{hand.nd,hr},c,i}+{\text{SOIL}}_{\text{hand.d,hr},c,i})+{\text{SA}}_{\text{hand,m}}*{\text{SOIL}}_{\text{hand,hr,m},i}+{\text{SOIL}}_{\text{obj,hr,c},i}\right)+{\text{SOIL}}_{\text{soil,day},c,i}$$

(2) $${\text{SOIL}}_{\text{hand.nd,hr},c,i}={\text{SL}}_{\text{c}}*\text{HF}.0_{\text{c}}*{\text{HM.nd}}_{\text{c},i}*{\text{HM.o}}_{\text{c}}*\text{HMTE}+{\text{SL}}_{\text{c}}*{\text{HF.po}}_{\text{c}}*{\text{HM, nd}}_{\text{c},i}*\left(1–{\text{HM.o}}_{\text{c}}\right)*\text{PTE}$$

(3) $${\text{SOIL}}_{\text{hand.d,hr},c,i}={\text{SL}}_{\text{c}}*\text{HF}.0_{\text{c}}*{\text{HM.d.episode}}_{\text{c},i}*{\text{HM.o}}_{\text{c}}*\text{HMTE}+{\text{SL}}_{\text{c}}*{\text{HF.po}}_{\text{c}}*{\text{HM.d.episode}}_{\text{c},i}*\left(1–{\text{HM.o}}_{\text{c}}\right)*\text{PTE}$$

(4) $${\text{SOIL}}_{\text{hand,hr,m},i}={\text{SL}}_{\text{m}}*{\text{HF.po}}_{\text{m},i}*{\text{HM.nd}}_{\text{m},i}*\text{PTE}+{\text{SL}}_{\text{m}}*{\text{HF.o}}_{\text{m},i}*{\text{HM.d.episode}}_{\text{m},i}*\text{HMTE}$$

(5) $${\text{SOIL}}_{\text{obj,hr,c},i}={\text{SL}}_{\text{obj}}*{\text{SAM}}_{\text{obj}}*{\text{OM}}_{\text{c},i}*\text{OMTE}$$

(6) $${\text{SOIL}}_{\text{soil,day,c},i}={\text{DIS}}_{\text{c},i}*{\text{SM}}_{\text{c},i}*{\text{SW}}_{\text{DI}}$$

### Ingestion of soil from mouthing hands

#### Load of soil on children’s hands (SL_c_) and mothers’ hands (SL_m_)

We modeled the load of soil on hands as independent of age. Based on our observational data [19], crawling children touched soil more frequently than walking children; however, the median number of times that children of different age groups touched the ground without touching other objects in between ranged from one to three. A study of adults pressing their index fingers onto a plate covered with dust found that after the first contact the hand was coated with 61% of the dislodgeable dust compared to 94% after the third contact [25], so we concluded that the amount of soil adhered to hands is predominantly determined by the initial contact. Consequently, we estimated the load of soil on the hands of children of all ages with the load of soil on the hands of children who provided hand rinse samples (median age: 36 months). Similarly, we estimated the load of soil on the hands of caregivers with the load of soil on the hands of caregivers who provided hand rinse samples.

To estimate the soil load on children’s hands, we divided the mass of soil rinsed from their hands by their estimated hand surface area. This estimated surface area is based on the child-specific linear trend between age in months and hand surface area calculated from anthropometric data collected during WASH Benefits [22] (see the SI for details). When a child’s hand is pressed into the soil, as in crawling, soil does not uniformly coat the entire surface of the hand, but rather primarily coats the fingertips, thumb, and heel of the hand [25, 26]. While these portions of the hand are frequently mouthed, there are also other portions of the hand that are frequently mouthed [23]. There are insufficient data to determine the fraction of the contaminated portion of the hand that enters the mouth in each hand-to-mouth contact, so we estimated soil ingested in each hand-to-mouth contact by modeling both soil and mouthed surface area as evenly distributed over the hand. The surface area of the hand mouthed is different for oral and peri-oral contacts [23].

### Direct ingestion of soil

#### Fraction of children directly ingesting soil (DIS_c,*i*_)

Our observations, which covered approximately 50% of children’s waking hours, suggested that in 1 day 22% of children 3–5 months old, 56% of children 6–11 months old, 56% of children 12–23 months old, 15% of children 24–35 months old, and 0% of children 36–47 months old directly consume soil during a typical day [18, 19]. Our observational data differed from the caregiver-reported prevalence of daily soil ingestion by <10%. Averaging the 1-day recall periods, 0% of children 3–5 months were reported ingesting soil, compared to 53% of children 6–11 months old, 50% of children 12–23 months old, and 27% of children 24–35 months old (WASH Benefits, unpublished data). Mothers are frequently busy and may not observe or accurately recall direct soil ingestion events, so we used our observational data to model direct soil ingestion. However, instead of using the observation that 0% of the 6 children 36–47 months old ingested soil, we used 20%, the mean of the upper and lower 95% confidence interval of the observed data calculated using the Wilson score interval [27].

#### Frequency of directly ingesting soil by licking soil or placing it in the mouth, among mouthers only (SM_c,*i*_)

We calculated the frequency of licking soil or placing soil in the mouth among children that performed these behaviors. A simulated child either consumes soil directly, with an ingestion frequency based on observed frequencies of those children who consumed soil, or does not, with an ingestion frequency of zero.

#### Mass of soil directly ingested by licking soil or placing it into the mouth (SW_DI_)

We reviewed the videos to observe the number of times children placed soil in their mouths. Children were observed placing soil in the mouth and then removing it, placing soil in the mouth and ingesting it, and licking earthen surfaces (i.e. the windowsill of an earthen home). Soil that children placed in the mouth was approximately the size of a grain of rice, which ranges in size from 0.02 to 0.07 cm^3^. Given a bulk density of 1.0 g/dry cm^3^ soil [28], we estimated that these ingested pieces of soil were 20–70 dry mg. We estimated the amount of mass removed from licking an earthen surface to be 7 mg/lick based on a study that found three licks of a palm coated with soil removed 22.1 wet mg of soil [29]. To estimate the quantity of soil ingested per direct ingestion event, we used these values to define a triangular distribution with a minimum of 7 mg, a maximum of 70 mg, and a mode of 20 mg. To more realistically represent the variation in the amount of dry soil ingested per direct soil ingestion event, we approximated this triangular distribution with a beta distribution [30], beta (5.3, 158.6).

### Transfer efficiencies

#### Hand-to-mouth transfer efficiency (HMTE) and object-to-mouth transfer efficiency (OMTE)

Hand-to-mouth transfer efficiency is the fraction of residue that transfers from the skin to the mouth via saliva and mechanical action of the tongue. In a study estimating transfer of lead from fingertips into the mouth, six volunteers handed lead fishing weights with three of their fingertips, then pressed their fingertips into pools of their own saliva [31]. The resulting transfer efficiency of 24% (sd = 3.3%) did not incorporate transfer due to sucking on fingers or the friction of the tongue but suggests that 24% is an appropriate empirically derived lower bound for the transfer of soil from skin to mouth.

To estimate the hand-to-mouth transfer efficiency, we used maximum likelihood estimation to approximate a triangular distribution (min = 0.24, max = 1.00) the distribution beta (5.2, 2.6). More information on the hand-to-mouth transfer efficiency is provided in the SI. Given the lack of data on hand-to-mouth versus object-to-mouth hand-to-mouth transfer efficiency, we used the same distribution for both parameters.

#### Peri-oral transfer efficiency (PTE)

Peri-oral transfer efficiency is the efficiency with which residue transfers from skin to the lips or area around the mouth and then into the mouth. The transfer efficiency of saffron powder from hand to lips was 37% (90th percentile: 91%) and from lips to mouth was 38% (90th percentile: 82%) [26]. We approximated a triangular distribution (min = 0.01, max = 0.91 × 0.82, mean = 0.37 × 0.38) with a distribution beta (5.2, 2.6).

### Other parameters

The frequency of hand-to-mouth (HM.nd_c__,i_, HM.nd_m_) and object-to-mouth contacts (OM_c__,i_) have been described [18, 19]. We used the same video observations to assess the frequency of contacts with caregivers’ hands and contacts with hands during eating episodes (HM.d.episode_c__,i_, HM.d. episode_m_). We observed that hand recontamination was infrequent while eating, so we modeled the amount of contamination transferred during eating-related hand-to-mouth contacts differently from hand-to-mouth contacts that were unrelated to eating. We assumed that when the child is not eating, there is complete recontamination of hands between each hand-to-mouth contact. This applies to both contacts with children’s own hands and the hands of their caregivers. However, for hand-to-mouth contacts that occur during eating, both children’s hands and caregivers’ hands are often holding food and rarely touch soil between two successive hand-to-mouth events. In observed episodes of the child self-feeding (*n* = 703), the child touched soil in 38 (5%) of episodes and touched soil more than once in only 2% of episodes. As such, we modeled contamination on the hand transferring to the mouth as one single contact per eating episode rather than during each hand-to-mouth contact that occurred while eating. Detailed descriptions of these methods are provided in the SI, as are methods for calculating the surface area of children’s hands (SA_c_) and mother’s hands, the fraction of child’s hand mouthed per oral contact (HF.o_c__,i_) and peri-oral contact (HF.po_c,*i*_), the fraction of caregiver’s hand mouthed per oral contact (HF. o_m,*i*_) and peri-oral contact (HF.po_m,*i*_), the load of soil on objects (SL_o_), the mouthed surface area of objects put into the mouth (SAM_o_), and the duration that children are awake per day (awake_c,*i*_) [32].

### Sensitivity and other analyses

To conduct sensitivity analyses, we set all parameters to their median values except for the parameter of interest, which was set to its 25th percentile for the first model run and 75th percentile for the second model run. For each parameter, the magnitude of the ratio between the results of the 75th percentile run and 25th percentile run indicated the influence of the parameter on the model. We present the age-specific ratio of the 75th to 25th percentile results when the standard deviation of the ratio for the age group differs more than 10% from the mean ratio for all age groups. We also changed the selected distributions of specific model parameters and eliminated some of the parameters to assess how the resulting estimates of soil ingestion compare to previous assessments. Finally, we calculated the contribution of each pathway to the total quantity of soil ingested by assessing the percent that each pathway contributed to the total in each simulation and averaging these values after weighting by the log_10_ of the total amount of soil ingested. We report geometric means, unless noted otherwise.

## Results

**Fig. 1:**
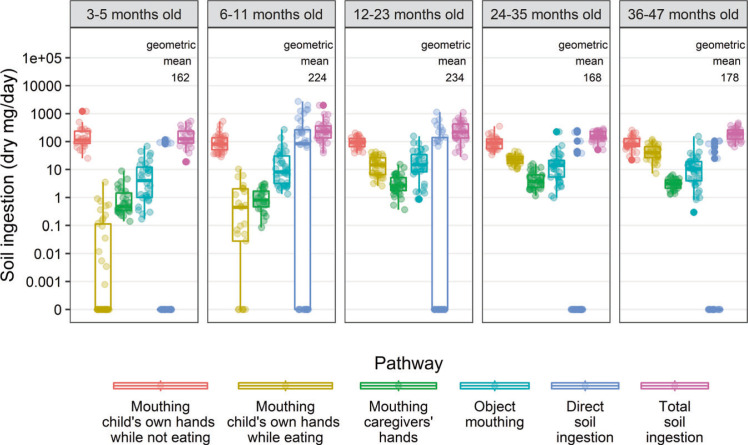
Modeled soil ingestion among young children in rural Bangladesh. In the boxplots of soil ingestion across multiple pathways, the median is denoted by the line in the middle of the box, with the bottom of the box marking the 25th percentile and the top of the box marking the 75th percentile. The whiskers extend past the box to 1.5 times the range between the 25th and 75th percentile

We estimate that children 3–5 months old ingested a geometric mean of 162 dry mg of soil/day. Modeled soil ingestion was almost 1.5 times higher than among children 6–11 months old (224 dry mg/day) and children 12–23 months old (234 dry mg/day). Older children consumed a geometric mean estimate of 168 dry mg/day for children 24–35 months old and 178 dry mg/day for children 36–47 months old (Fig. 1).

**Fig. 2:**
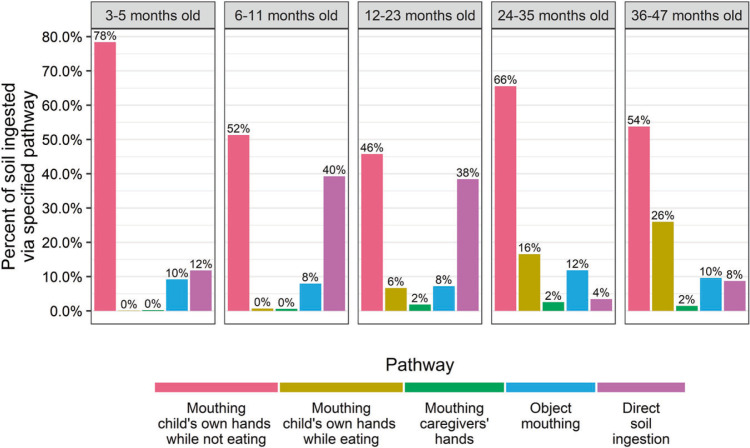
Relative contribution of multiple pathways to modeled soil ingested by young children in rural Bangladesh

The predominant pathway of modeled soil ingestion was children placing their own hands in their mouths unrelated to eating, which contributed 46–78% of total soil ingestion across age groups (Fig. 2). Among children 6–23 months old, direct consumption of soil was as substantial as soil ingestion through mouthing hands (38–40%). For other age groups, direct consumption of soil was a minor pathway (4–12%) of total soil ingestion. Across all age groups, object-mouthing contributed 8–12% of the total. Soil ingested due to mouthing hands while eating was a more prominent pathway for older children, contributing 16–26% for children ≥24 months. Mouthing caregivers’ hands contributed ≤2% of total ingestion across all age groups.

**Fig. 3:**
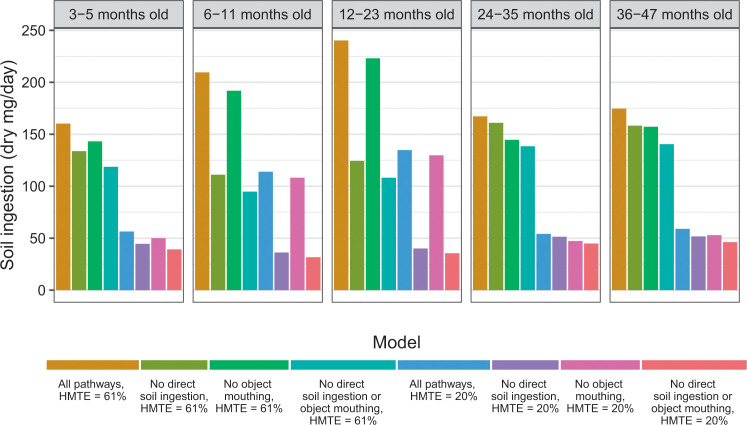
Effect of altering model parameters on modeled soil ingestion. Modified parameters were the distribution used for the hand-to-mouth transfer efficiency (HMTE), eliminating the contribution of direct ingestion of soil and/or eliminating the contribution of soil indirectly ingested through placing objects in the mouth

Our estimates for soil ingestion are substantially higher than for children in high-income countries (Table 2). To more accurately compare our results with those of other studies, we evaluated both the effect of the hand-to-mouth transfer efficiency parameter and exclusion of particular ingestion pathways. When we lowered the hand-to-mouth transfer efficiency from a mean of 61% to a mean of 20%, mimicking the hand-to-mouth transfer efficiency used in a study of U.S. children, our estimates dropped to 57–120 dry mg/day. When we also eliminated the direct ingestion pathway, similar to the same study of U.S. children that estimated an ingestion geometric mean of 36 dry mg soil/day for children 36–72 months old [7], the soil ingestion rate for children in our study dropped to 38–52 dry mg/day. When the distribution of the hand-to-mouth transfer efficiency parameter for Bangladesh was used and the direct ingestion pathway and object-to-mouth pathway were eliminated, similar to a model that estimated Canadian children <59 months old ingested 36–61 dry mg soil/day [13], the estimated geometric mean soil ingestion for children 3–47 months old in our study dropped to 97–141 dry mg/day (Fig. 3). Accounting for different hand-to-mouth transfer efficiencies and pathways of ingestion in activity pattern studies resulted in similar estimates for children in Bangladesh and the U.S. [7], but not Canada [13].

**Table 2:** Summary of studies on young children’s ingestion of soil and dust, ordered from highest to lowest estimated ingestion rate

Study	*N*	Location	Age group	Estimated ingestion of soil and/or dust [mg/day]	Method	Source				
						Soil-to-mouth	Hand-to-mouth	Object-to-mouth	Soil	Dust
Ngure [21]	3	Zimbabwe	1 year	mean: 21,250	activity pattern	X	X		X	
Day [11]	theoretical	England	not given	10–1000	activity pattern			X	X	
Wong [42]	52	Jamaica	0.3–14 years	470 for children 0.3–7.5 years old; 58 for children 1.8–14 years old	tracer	X	X	X	X	X
This study	simulation	Bangladesh	1–<2 years	geometric mean: 234 (geo sd 2)	activity pattern	X	X	X	X	X
This study	simulation	Bangladesh	0.5– <1 years	geometric mean: 224 (geo sd 2)	activity pattern	X	X	X	X	X
This study	simulation	Bangladesh	3– <4 years	geometric mean: 178 (geo sd 2)	activity pattern	X	X	X	X	X
This study	simulation	Bangladesh	2– <3 years	geometric mean: 168 (geo sd 2)	activity pattern	X	X	X	X	X
This study	simulation	Bangladesh	3— 5 months	geometric mean: 162 (geo sd 2)	activity pattern	X	X	X	X	X
Hogan [35]	142	USA	3–5 years	135	biokinetic	X	X	X	X	X
Hogan [35]	164	USA	1–3 years	135	biokinetic	X	X	X	X	X
Binder [36]	65	USA	1–3 years	108	tracer	X	X	X	X	X
Lepow [9]	22	USA	2–6 years	100	activity pattern		X	X	X	X
Bothe [37]	8	Germany	1–2 years	100	tracer	X	X	X	X	X
van Wijnen [17]	468	Netherlands	1–5 years	geometric mean: 0–200	tracer	X	X	X	X	X
von Lindern [34]	model	USA	1–2 years	mean: 93	biokinetic	X	X	X	X	X
EPA [14]	NA	USA	1–2 years	mean: 90 (40 soil and 50 dust)	recommendation					
Hogan [35]	38	USA	<1 year	85	biokinetic	X	X	X	X	X
von Lindern [34]	model	USA	0.5–1 year	mean: 84	biokinetic	X	X	X	X	X
EPA [14]	NA	USA	0.5–1 year	mean: 70 (30 soil and 40 dust)	recommendation					
Ozkaynak [7]	simulation	USA	3–6 years	mean: 68	activity pattern		X	X	X	X
von Lindern [34]	model	USA	2–3 years	mean: 67	biokinetic	X	X	X	X	X
von Lindern [34]	model	USA	4–5 years	mean: 65	biokinetic	X	X	X	X	X
von Lindern [34]	model	USA	3–4 years	mean: 62	biokinetic	X	X	X	X	X
Wilson [13]	simulation	Canada	0.5–5 years	mean: 61 (20 (sd 26) soil and 41 (sd 71) dust)	activity pattern		X		X	X
EPA [14]	NA	USA	2–6 years	mean: 60 (30 soil and 30 dust)	recommendation					
Stanek [33]	47	USA	3–4 years	median: 57.0, mean: 32.2 (se 22.3)	tracer	X	X	X	X	X
Clausing [38]	18	Netherlands	2–4 years	mean: 56	tracer	X	X	X	X	X
Lin [47]	177	China	2.5–12 years	median: 52, mean: 74	tracer	X	X	X	X	X
EPA [14]	NA	USA	0–0.5 years	mean: 40 (20 soil and 20 dust)	recommendation					
Wilson [13]	simulation	Canada	0–0.5 years	mean: 36 (0 soil and 36 (sd 130) dust)	activity pattern		X		X	X
Bothe [37]	23	Germany	2–7 years	35	tracer	X	X	X	X	X
Bothe [37]	7	Germany	<1 year	22	tracer	X	X	X	X	X
Stanek [33]	55	USA	2–3 years	median: 21.9, mean: 20.6 (se 24.0)	tracer	X	X	X	X	X
Duggan and William [12]	theoretical	England	not given	20	activity pattern		X		X	
Chien [39]	50	Taiwan	0.5–3 years	mean: 9.6 (sd 19.2)	tracer	X	X	X	X	X
Stanek [33]	39	USA	1–2 years	median: 9.2, mean: 3.8 (se 11.8)	tracer	X	X	X	X	X

Estimated soil ingestion among non-geophageous children less than 5 years old, by study and method

The parameters that most influenced soil ingestion estimates varied between age groups (Table S1). For children 6–23 months old, the frequency of direct ingestion of soil was the most influential model parameter. The model result using the 75th percentile (≈7–8 times/day) yielded estimates four times higher than the model using the 25th percentile (0 times/day). For children 3–5 or ≥24 months old, the frequency of direct soil ingestion was not an influential parameter because both the 25th and 75th percentile values were 0 times/day. For children 3–5 or ≥24 months old, the model was most sensitive to variation in the load of soil on children’s hands. For example, for children 36–47 months old, the 75th percentile estimate for the load of soil on one hand (0.13 dry mg/cm^2^) resulted in a total quantity of ingestion that was four times greater than when the model was run with the 25th percentile estimate (0.03 dry mg/cm^2^). For these children the frequency of child hand-mouthing while not eating also altered the estimated mass of soil ingested by 2–6 times. Variation in the fraction of hand-to-mouth contacts that involved part of the hand entering the mouth (an oral contact) versus touching the lips (a peri-oral contact) and variation in the fraction of the child’s hand that entered the mouth during an oral contact also had the potential to double the estimate.

## Discussion

Children in our study ingested an estimated 162–234 dry mg soil/day, substantially higher than estimates reported by empirically based studies in the U.S. [7, 33–36], Canada [13], Germany [37], the Netherlands [17, 38], and Taiwan [39]. Our model accounted for direct consumption of soil, which previous assessments using activity pattern models have not included [7, 12, 13]. Young children across the globe have been reported to directly consume soil [5, 18–20, 40–43]. However, the amount of soil consumed by children each time they directly place soil into their mouths has been poorly quantified. While the mass of soil ingested per soil ingestion event was not identified as a highly influential parameter in our model, this is likely because of its narrow distribution (a 75th percentile of 40 mg and 25th percentile of 22 mg) in our dataset. As our results identify the frequency of direct soil ingestion as an influential model parameter and the mass of soil ingested per event has very weak empirical foundation, further research into both could substantially improve the accuracy of soil ingestion estimates.

Our soil ingestion estimates for children 36–47 months old are nearly double those determined in an activity patterns model of soil ingestion among children in the U.S. [7], in part due to that model’s use of a low hand-to-mouth transfer efficiency; the mean was less than half of the lower bound for the same parameter used in an assessment of children’s exposure to arsenic [44]. The mean is also less than half of other studies’ modal [13] and point estimates [45, 46] of efficiency, and one-third of the median efficiency used in this study. Further research on the transfer efficiencies of soil into the body from hand-to-mouth and object-to-mouth contacts could improve the accuracy of soil ingestion estimates.

In high-income countries, recent tracer studies have estimated that children ingest 9–57 dry mg soil/day (with one estimate for German children 1–2 years old at 100 dry mg/day [37]). Tracer studies account for direct and indirect ingestion of soil, indicating that our results may not be due to an omission of variables in other activity pattern studies but rather reflect that children in low-income settings such as rural Bangladesh do ingest substantially more soil than children in high-income settings. This may be due to a high prevalence of direct soil ingestion on a daily basis and frequent contacts with soil both outdoors and indoors (via earthen floors) that result in high soil loads on hands that are infrequently washed.

This study contributes to the sparse literature on soil ingestion among children in lower-income countries. Of the three published estimates of soil ingestion in lower-income countries, our estimates are 3–5 times higher than those for children in mainland China [47], within the range of estimates for institutionalized children in Jamaica [42], and substantially less than estimates for children in Zimbabwe [21] (Table 2). The authors of the study in Zimbabwe rubbed soil on a children’s hands to estimate that soil adhesion on heavily soiled hands was 250 wet mg/hand [21] whereas we rinsed children’s hands following normal daily activities and estimated a mean soil adhesion of 11.5 dry mg/hand. The accuracy of risk assessments focused on soil-borne hazards could be improved with rigorous quantification of the amount of soil ingested per direct soil ingestion event in a range of settings and among children of different age groups.

Our model has several limitations. The environmental contact data for children <12 months old was primarily based on data from a structured observation of children’s behaviors, which is likely undercounts environmental contacts compared to video observation, so results for these age group may be underestimated. The degree of undercounting/underestimation could be evaluated in future studies. Additionally, there is only environmental contact data from six children 36–47 months old, so results for this age group are less generalizable than those for other age groups. Literature values used for model parameters may be based on soil with different characteristics than the soil in Bangladesh.

We assumed that children of all ages have the same load of soil on their hands. We based this assumption on the maximal loading of soil on hands after fewer than five consecutive contacts with the hand and the ground. However, if younger children have an average load of soil on their hands that is higher than older children due to more frequently reaching maximal loading or other qualities that result in higher maximal load, then our model underestimates their consumption of soil due to hand-mouthing. Conversely, younger children may have lower loads of soil on their hands than older children because they are bathed or have their hands wiped more frequently, resulting in overestimates of their consumption due to hand-mouthing.

We did not account for events that remove soil from children’s hands, such as handwashing and bathing, because such removal events were infrequently observed in this setting. In a structured observation of children 3–5 years old, both hands were washed with water only in 2% of opportunities before eating; both hands were washed with soap and water in 6% of opportunities after defecation [48]. Our hand rinse samples were collected throughout the day and not following any particular event (e.g. handwashing or playing in the dirt), so we expect they are representative of the typical mass of soil on child and caregiver hands. However, if a child’s hands are washed and remain relatively clean during a substantial portion of the contacts between children’s hands and their mouths, we may have overestimated that quantity of indirect soil ingestion due to hand-to-mouth contacts. We could account for handwashing events by assessing the number of hand-to-mouth contacts that occur before contaminant load on the hand returns to pre-washed levels. We could then subtract this number from the total number of hand-to-mouth contacts before multiplying by contaminant load to estimate intake.

We assumed that *E. coli* on the sampled ball was attached to soil particles so used *E. coli* contamination on nonporous balls and in soil to estimate the contamination of soil on all objects that children put in their mouths. Estimating the amount of soil on hands by dividing the load of *E. coli* on hands by the concentration of *E. coli* in soil resulted in an underestimate in 12/19 observations, with a mean underestimate of 24 mg/2 hands and an overestimate in 7/19 times, with a mean overestimate of 89 mg/2 hands. For these 19 samples of hands, the overall mean difference between the estimated and measured amounts of soil on hand was 18 mg/2 hands compared to the empirically measured mean soil load of 26 mg/2 hands. This suggests that our method of using the load of *E. coli* on hands and objects was sometimes inaccurate, but not biased towards overestimating the mass of soil on objects and consequently the mass of soil ingested from objects in our model. Due to surface properties, objects that do not have the same surface type as the sentinel toys in this study (nonporous plastic) may carry more or less soil on their surfaces than the sentinel toys we measured. For example, children often mouthed their mother’s clothing, which we expect would not be as contaminated as a sentinel toy that was allowed to roll on the ground. However, objects such as leaves and sticks that are laying on the ground may be substantially more contaminated than the sentinel toys, which would result in an underestimation of soil ingestion from objects. The mass of soil on children’s hands was an influential parameter with regards to the total quantity of soil ingested. In this soil-laden setting, hands can be rapidly recontaminated because of frequent contacts with soil and objects [19]. Reducing the amount of soil transferred through hand-to-mouth contacts by washing children’s hands would require an impractical amount of handwashing. Efforts to reduce children’s exposure to soil, for example covering indoor floors and outdoor surfaces so they are not bare dirt, may more effectively reduce the load of soil on hands and objects and reduce associated disease risk [49].

We modeled soil ingestion among Bangladeshi children <48 months old using parameter values for exposure and environmental contamination levels from rural Bangladesh and find substantially higher levels of soil ingestion than among children in high-income countries. Children 6–23 months old consumed a geometric mean of 224–234 mg dry soil/day, while children 3–5 or ≥24 months old ingested 162–178 dry mg/day. Due to their direct consumption of soil, children 6–23 months old had the highest rates of soil ingestion. These results indicate that risks associated with soil faced by children in rural, low-income settings may be underestimated if risk assessors apply the estimates from high-income settings. Evaluation of the model compared to previous assessments also highlights the importance of considering all pathways of direct and indirect ingestion to accurately estimate children’s ingestion of soil. Strategies that prevent children from directly consuming soil, such as supervised play groups and flooring that reduces the accessibility to bare soil, merit further research on their ability to reduce soil exposure among children in this age group in low-income settings.
