# Supplementary figures and images for: Soil ingestion among young children in rural Bangladesh
Source: J Expo Sci Environ Epidemiol. 2019 Oct 31:1–12. doi: 10.1038/s41370-019-0177-7 (PMC7722350; doi:10.1038/s41370-019-0177-7)

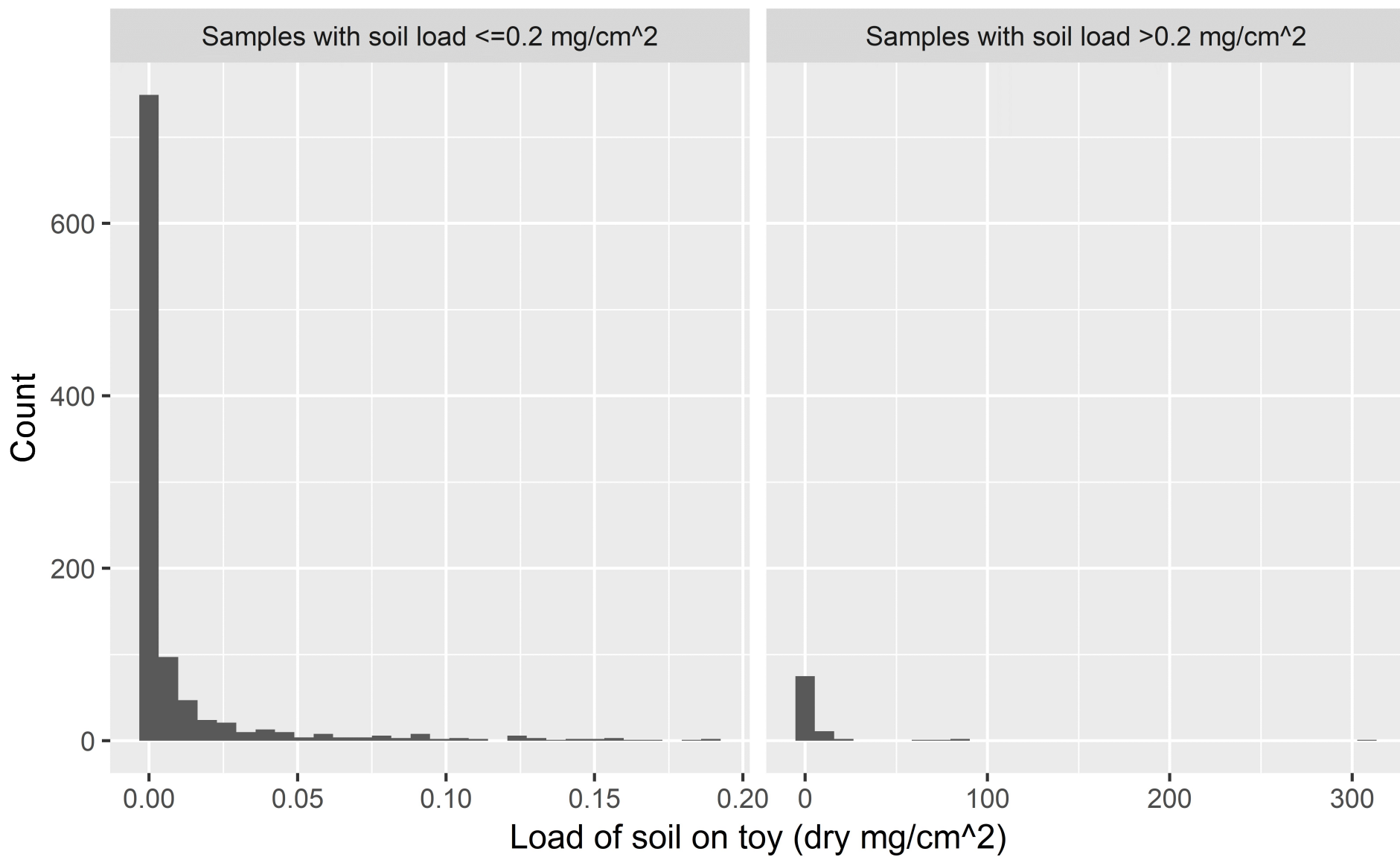

Supplement: Supplementary file 2 [file JESEE-2019-01777-s002.pdf]
